# Plants used by Brazilian communities of African descent for women's health

**DOI:** 10.1016/j.clinsp.2024.100513

**Published:** 2025-02-05

**Authors:** Letícia Francine Silva Ramos, Ananda Gomes de Sousa, Rebeca de Siqueira Amorim, Alan de Araújo Roque, Israel Luís Diniz Carvalho, Ana Laura Vilela de Carvalho, Maiara Bernardes Marques, Milena Evangelista dos Santos, Moan Jéfter Fernandes Costa, Luiza Rayanna Amorim de Lima, Pedro Henrique Sette-de-Souza

**Affiliations:** a Programa de Pós-Graduação em Saúde e Desenvolvimento Socioambiental, Universidade de Pernambuco (UPE), Campus Garanhuns, Garanhuns, PE, Brazil; b Faculdade de Odontologia, Universidade de Pernambuco (UPE), Campus Arcoverde, Arcoverde, PE, Brazil; c Herbário do Parque Estadual Dunas do Natal, Natal, RN, Brazil

**Keywords:** Ethnopharmacological knowledge, Natural products, Women vulnerability, Therapeutic uses, Quilombola women's

## Abstract

Quilombola women are primarily responsible for maintaining ethnopharmacological knowledge. Their knowledge is transmitted orally to their descendants, preserving cultural identity and ensuring the survival of Quilombola communities. Was conducting a scoping review on the use of medicinal plants by Quilombola women. Based on the PPC strategy (population, concept, and context) and the “Joanna Briggs Institute Reviewer's Manual”, this systematic scoping review sought to answer the guiding question: which medicinal plants are used for diseases that compromise women's health in Brazilian communities of African descent? The article has registered this research in the Open Science Framework available at osf.io/eb2nc, at identifier DOI https://doi.org/10.17605/OSF.IO/5N24Y. Using the search keys, 888 studies were found. After reading the titles and abstracts, 38 studies moved on to the next stage. During the reading of the texts in full, 32 were excluded for not meeting all the inclusion criteria and six studies were included in the research. In all, 44 plants, belonging to 23 families, were mentioned in the studies. The plants reported by the study have a plurality in their therapeutic use, the form of preparation, and part of the plant used. Regarding women's health, plants were recorded for sexual, menstrual, and postpartum care. The appreciation and recognition of this ancestral knowledge contribute not only to Women's health but also to strengthening the identity and rescuing the cultural roots of these communities.

## Highlights

• Ethnopharmacological knowledge is passed down through generations by women in the community, who use plants especially for sexual and postpartum care.

## Introduction

Quilombola communities, considered traditionally mostly of African ancestry, are historically marginalized groups whose trajectory goes back to Brazil's colonial and slave past. They are territories of struggle and resistance and are distributed throughout Brazil, especially in rural areas.^1^ The context of extreme socioeconomic inequality of these communities in relation to other Brazilian populations places them in difficulties of access to their basic rights, such as health.^2^

In these Quilombos, women are often holders of traditional knowledge, including knowledge related to medicine, cooking, agriculture, and crafts, among others. This knowledge is passed down from generation to generation and plays an important role in preserving cultural identity and ensuring the survival of communities.^3^

However, women often face challenges in disseminating this traditional knowledge, including lack of recognition and appreciation by government authorities and institutions, loss of land and natural resources that affect their traditional practices, and gender discrimination that excludes them from positions of power and influence.^4^ Therefore, it is important to ensure the protection and promotion of traditional knowledge held by women, as well as their rights and equal participation in decisions affecting their communities and their cultural heritage.^4^

Women's knowledge regarding health care permeates the use of medicinal plants in the face of illness and various diseases. All this knowledge may be associated with the low accessibility of communities to the Unified Health System (SUS), due to social marginalization and geographical distance. It is in this sense that studies on the use of medicinal plants and their relationship with women's health gain prominence. Among the main pathophysiological aspects studied are changes and pain during the menstrual cycle.^5^ Aspects related to pregnancy^6^ and sexual dysfunction.^7^

However, even with the increase in research on the use of medicinal plants and women's health, there are few studies that relate the use of medicinal plants to the health of Quilombola women in Brazil, especially in high-impact journals. Therefore, this work aims to preserve and value the traditional knowledge of these communities, promoting the dissemination of ancestral practices of care and well-being specific to the needs of women in this cultural context.

## Methodology

### *Protocol and registration*

This study is a systematic scoping review, which followed all the recommendations proposed by PRISMA extensions for Scoping Reviews (Preferred Reporting Items for Systematic Reviews and Meta-Analyses). The authors have registered this research in the Open Science Framework available at osf.io/eb2nc.

### *Eligibility criteria*

Based on the PPC strategy (population, concept, and context) and the “Joanna Briggs Institute Reviewer's Manual”,^8^ this systematic scoping review sought to answer the guiding question: which medicinal plants are used for diseases that compromise women's health in Brazilian communities of African descent?

To this end, the following inclusion criteria were applied in the selection of studies: (i) To be original and peer-reviewed articles; (ii) Articles that identified through the deposit of exsiccates which plants the Quilombola communities used to treat health problems; (iii) To be written in Portuguese, English or Spanish; (iv) Without restrictions on the year of publication.

Studies that did not specify that their respective samples were of individuals declared Quilombolas were excluded, in view of the legal recognition of these individuals. In addition, reviews, book chapters, books, theses, dissertations, monographs, letters to the editor and case reports were excluded.

### *Information source and search*

The search algorithm construction process was based on the terms related to the inclusion criteria. The search algorithm was: (“Ethnic Groups” OR “Quilombolas”) AND (“Plants, medicinal” OR “Phytotherapy” OR “Medicine, traditional” OR “Ethnobotany”) AND (“Surveys” OR “questionnaires”). The databases used for the search were: *Biblioteca Virtual de Saúde* (BVS), PubMed, EMBASE, Scopus, Web of Science, Science Direct, and SciELO. Once the articles were included in these databases, a manual search was performed in the reference lists from Google Scholar, appropriating the gray literature to expand the virtual search scope.

### *Selection of evidence sources*

The studies included in this review were selected by two independent blind reviewers (R.S.A.; A.G.S.) using the online Rayyan QCRI (RRID: SCR_017584) to analyze the articles found resulting from the applied search strategy. In case of inconsistencies, a third reviewer (A.L.V.C.) was involved in the process.

The Kappa test generated an intra-examiner (100% agreement; kappa 1.00, 95% CI; 95.74% agreement; kappa 0.91, 95% CI; 100% agreement; kappa 1.00, 95% CI, for examiners 1, 2 and 3, respectively) and inter-examiner (96.25% agreement; kappa 0.93, 95% CI) coefficient. The reviewers initially removed duplicates. Subsequently, the papers were evaluated sequentially according to their title and abstract, following the related inclusion and exclusion criteria.

### *Data charting process*

The information extracted from the articles was grouped as follows: publication data (authors; year of publication; objective), research data (location; design; sample), and plants used according to the health problem of women (scientific name; form of preparation; health problem). The main results related to these aspects and the guiding question of the study were synthesized, according to the guidelines of the “JBI Evidence Synthesis Manual”.^9^

### *Critical appraisal of individual sources of evidence*

The risk of bias analysis was performed using Cochrane's RoB2 bias analysis platform. The analysis is separated into selection bias, performance bias, detection bias, attrition bias, reporting bias, and other conditions found.

### *Synthesis of results*

All the data were analyzed in a Microsoft Office Excel 2019 spreadsheet. A quantitative and descriptive survey of the data analyzed was conducted.

## Results

**Fig. 1:**
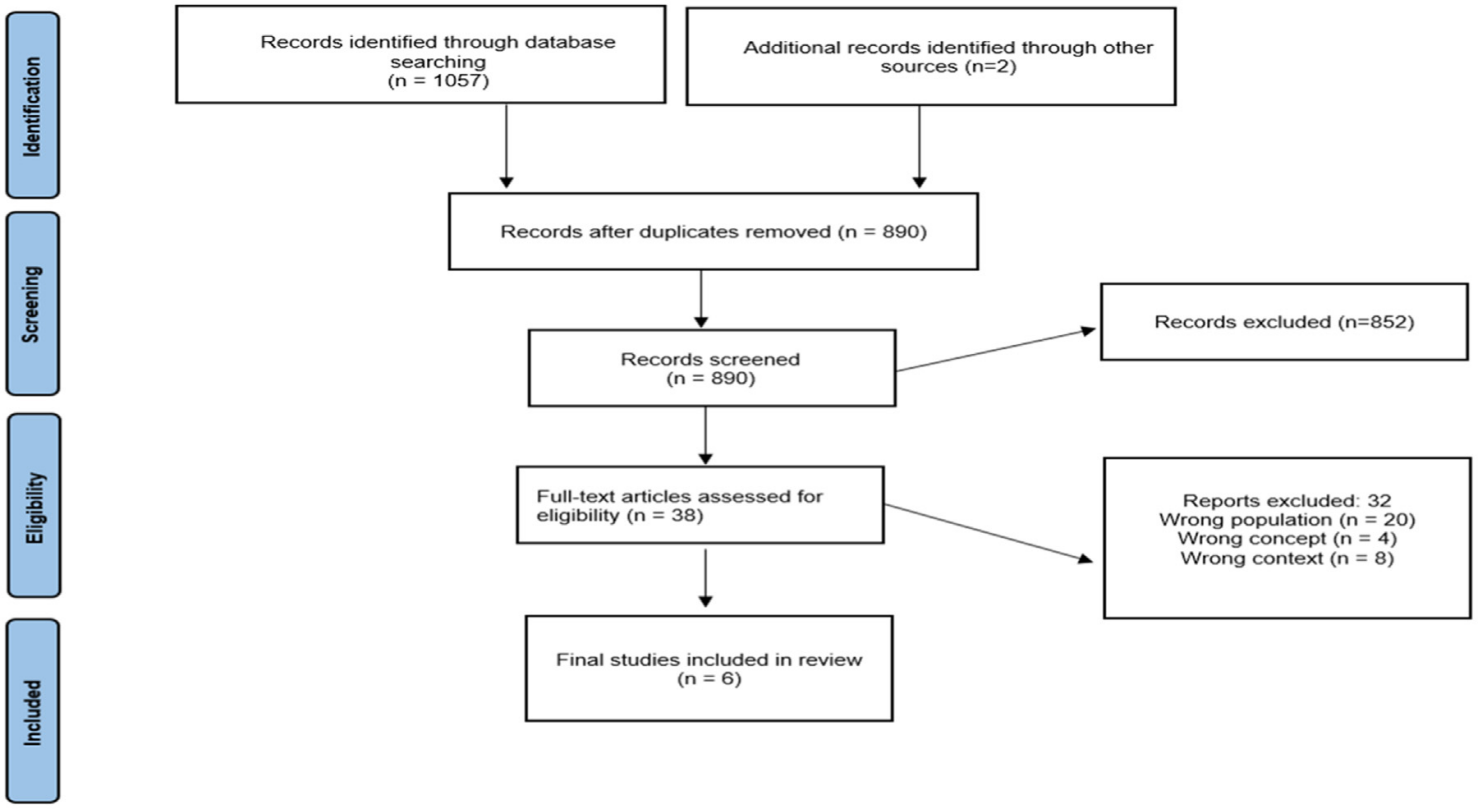
Flowchart detailing the phases of the research. (Source: Authors).

Through the search tools, 888 studies were found. After reading the titles and abstracts, 38 studies were eligible for the next step. After reading the full texts, 32 were eliminated because they did not meet all the inclusion criteria, totaling 6 studies that were included in the research. The details of the research phases are contained in Fig. 1.

**Fig. 2:**
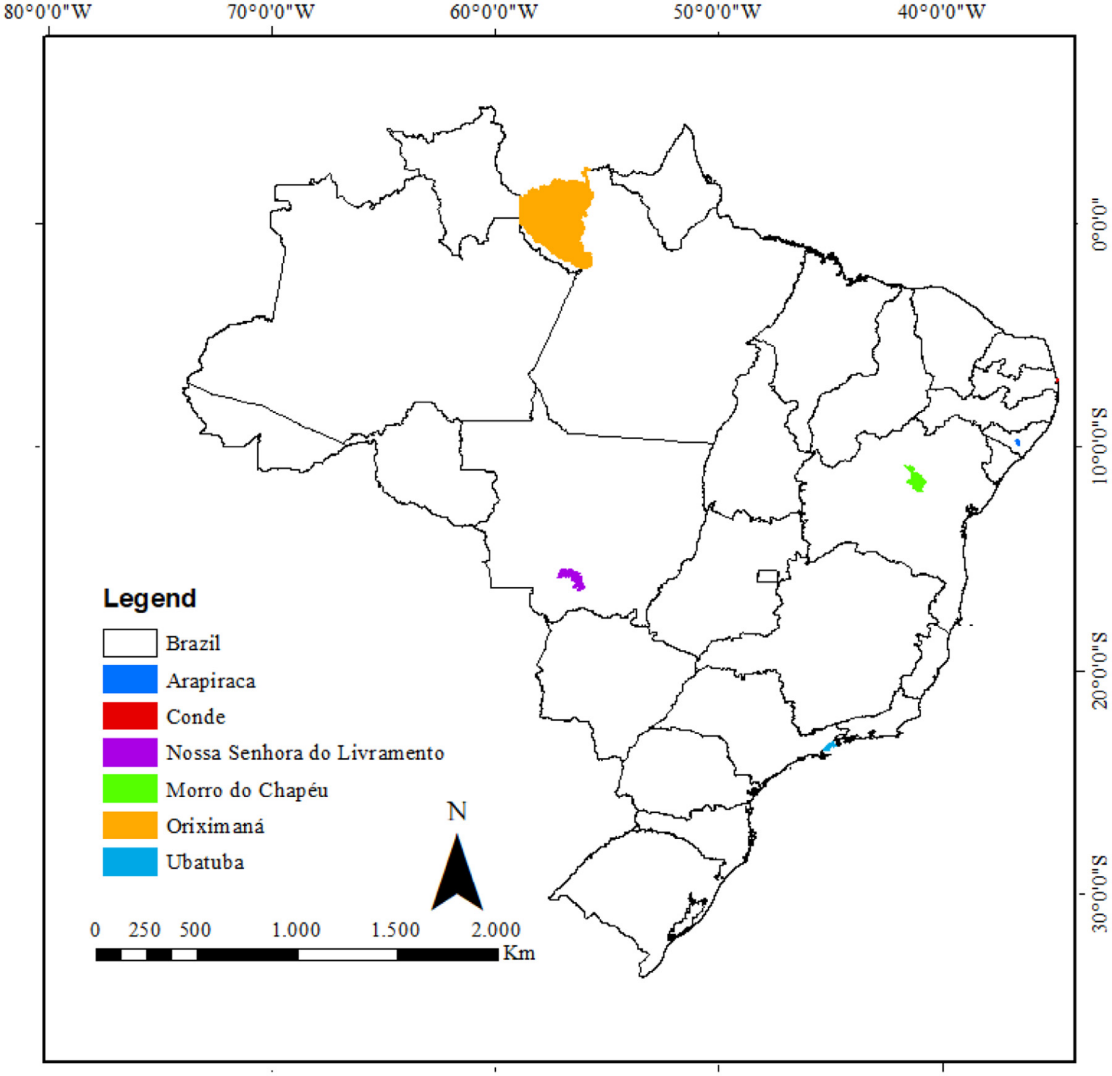
Regions with the most plants used by Quilombola communities for Women's health. (Source: Authors).

The articles found have a diverse publication period, between the years 2007 and 2022. In all, 44 plant species belonging to 23 families were reported. No identical plant species with different popular names were found. There was a greater report of plants used for women's health by communities in the Brazilian Northeast (3), Center-West (1), North (1), and Southeast (1) (Fig. 2).

The plants reported by the Quilombola communities studied have a plurality in their therapeutic use, a form of preparation, and part of the plant used. Regarding women's health, plants were recorded for sexual, menstrual, and postpartum care. Most of the plants were cited in the aid of inflammatory processes,^10^ specifically of the parturient/puerperium woman. However, the plants reported have other functions in the pregnancy process, including: stimulating uterine contraction^10^^,^^11^ cleaning remains of the uterus,^10^ postpartum,^10^^,^^11^^,^^12^ fertility,^10^ and abortifacient.^10^^,^^13^^,^^14^

Moreover, they have therapeutic potential in the menstrual cycle: acting on menstrual cramps,^10^^,^^13^^,^^14^ and on menstrual regulation^10^^,^^11^^,^^15^ three of them to increase and one to decrease the flow. It also acts to relieve menopausal symptoms, including heat.^11^ Among the functionalities found are: vaginal discharge,^10^ uterus,^10^ women's pain,^10^ women's disease,^10^ and ovarian infection.^11^

Concerning the part of the plants that are used for phytotherapeutic purposes, leaves^10^, ^11^, ^12^, ^13^, ^14^, ^15^ were the most reported, 29 times in total, followed by: bark,^10^^,^^14^ complete plant,^10^^,^^14^ seeds,^10^^,^^14^ flower,^10^^,^^13^ root,^10^ bulb,^10^ fruit,^10^ stem,^11^ and aerial part.^12^

Some of the species found have more than one form of preparation, among the most used are: decoction,^10^^,^^14^ infusion,^10^^,^^11^ maceration in alcohol,^10^^,^^15^ juice,^10^^,^^14^ maceration,^10^^,^^15^ not informed,^11^, ^12^, ^13^ and *in natura*.^10^ Detailed information is shown in Table 1.

**Table 1:** Ethnobotanical survey of medicinal plants from Quilombola communities applied to women's health (Source: Authors, 2023).

**Author (year)**	**Scientific name**	**Trivial name**	**Family**	**Parts used**	**Therapeutic indication**	**Form of Preparation**
Barboza da Silva et al. (2012)	*Abarema cochliacarpos* (Gomes) Barneby &J.W. Grimes	Barbatimão	Fabaceae	Bark	Reduce inflammation after childbirth;	Maceration; Maceration in alcohol; Decoction
	*Allium ascalonicum* L.	Cebola-branca	Amaryllidaceae	Bulb	Increase contraction; Avoid inflammation after childbirth	Decoction; Maceration in alcohol
	*Allium cepa* L.	Cebola	Amaryllidaceae	Bulb	Induce period; Removes postpartum uterine fragments	Decoction; Maceration in alcohol
	*Amburana cearensis* (Allem.) A.C. Smith	Imburana/emburana/umburana	Fabaceae	Bark; Leaves; Seeds	Menstrual cramps	Decoction; Infusion
	*Artemisia* sp.	Losna	Asteraceae	Leaves	Menstrual cramps; Prevent inflammation after childbirth	Decoction; Infusion; Maceration in alcohol
	*Artemisia vulgaris* L.	Artemijo	Asteraceae	Leaves	Removes postpartum uterine fragments	Juice; Infusion; Maceration in alcohol
	*Bidens pilosa* L.	Carrapicho	Asteraceae	Seeds; Leaves	Avoid inflammation after childbirth	Decoction; Infusion; Maceration in alcohol
	*Bixa orellana* L.	Urucum	Bixaceae	Seed; Leaves	Menstrual cramps; Ovary pain	Maceration; Infusion
	*Boerhaavia coccinea* Willd.	Pega pinto	Nyctaginaceae	Whole plant	Avoid inflammation after childbirth	Maceration in alcohol
	*Chamaecrista cytisoides* var. *blanchetti* (Benth) H.S. Irwin & Barneby	Rompe gibão	Asteraceae	Leaves	Inflammation in women	Decoction
	*Chenopodium ambrosioides* L.	Mastruz	Amaranthaceae	Whole plant	Woman's pain; Inflammation in women	Decoction; Infusion; Juice
	*Chrysanthemum parthenium* (L.) Bernh.	Macela/Macela galega	Asteraceae	Leaves; Flower	Menstrual cramps; Puerperal woman/ Removes postpartum uterine fragments	Decoction; Infusion; Maceration in alcohol
	*Cinnamomum zeylanicum* Breyn.	Canela	Lauraceae	Bark	Uterus; Avoid inflammation after childbirth	Decoction; Maceration
	*Coutareae hexandra* (Jacq.) K. Schum.	Quina	Rubiaceae	Bark	Abortifacient	Decoction; Maceration in alcohol; In Natura
	*Gossypium herbaceum* L.	Algodão	Malvaceae	Fruit; Leaves	Inflammation in women	Juice; Decoction
	*Handroanthus impetiginosus* (Mart. ex DC.) Mattos	Pau d'arco roxo	Bignoniaceae	Bark	Inflammation in women	Maceration
	*Mangifera indica L.*	Manga espada	Anacardiaceae	Leaves	Women's diseases	Decoction
	*Mentha pulegium* L.	Peijo (poeijo)	Lamiaceae	Leaves	Removes postpartum uterine fragments	Decoction; Infusion; Maceration in alcohol
	*Mentha viridis* L.	Hortelã miúdo	Lamiaceae	Leaves	Increase menstruation; Uterus	Juice; Decoction
	*Momordica charantia* L	São caetano	Cucurbitaceae	Leaves	Inflammation in women	Decoction
	*Myracrodruon urundeuva* Allemão	Aroeira	Anacardiaceae	Leaves	Inflammation in women	Infusion; Decoction; Maceration
	*Myristica fragans* L.	Noz-moscada/Manuscada	Myristicaceae	Seed	Menstrual cramps; Uterus	Infusion; Maceration in alcohol; Decoction
	*Phyllanthus flaviflorus* (K.Schum. & Lauterb.) Airy Shaw	Quebra pedra	Euphorbiaceae	Whole plant	Inflammation in women	Decoction; Infusion
	*Plantago major* L.	Trançagem	Plantaginaceae	Whole plant	Inflammation in women; Avoid inflammation after childbirth	Decoction; Infusion; In Natura; Maceration in alcohol
	*Plectranthus amboinicus* Lour.	Hortelã grosso/Graúdo	Lamiaceae	Leaves	Increase menstruation; Inflammation in women; Discharge	Juice; Decoction
	*Plectranthus barbatus* Andr.	Boldo/Sete dores	Lamiaceae	Leaves	Menstrual cramps	Infusion; Decoction
	*Pluchea sagittalis* (Lam.) Cabrera	Quitoco	Asteraceae	Root; Leaves	Inflammation after childbirth	Decoction
	*Psidium guajava* L.	Goiabeira	Myrtaceae	Bark; Leaves	Avoid inflammation after childbirth	Decoction
	*Ruta graveolens* L.	Arruda	Rutaceae	Leaves	Increase contraction; Removes postpartum uterine fragments	Decoction; Maceration in alcohol
	*Senna occidentalis* (L.) Link	Fedegoso	Fabaceae	Leaves; Root; Flower	Inflammation or pain after childbirth	Infusion; Decoction
	*Solanum ambrosiacum* Vell.	Melancia da praia	Solanaceae	Whole plant	Discharge	Decoction
	*Solanum erianthum* D. Don	Caiçara	Solanaceae	Raiz	Discharge	Decoction
	*Sphagneticola trilobata* (L.) Pruski	Calêndula	Asteraceae	Whole plant	Inflammation in women; Menstrual cramps	Decoction; Infusion; Maceration in alcohol
	*Syzygium cumini* (L.) Skeels	Janelão/Jamelão	Myrtaceae	Leaves; Fruit	Inflammation in women	Decoction; Infusion; In Natura
	*Vitis aestivalis* (Bailey) B.L.Comeaux	Uva	Vitaceae	Leaves	Decrease menstruation	Decoction
Beltreschi et al. (2018)	*Lippia alba (*Mill.) N.E. Br. *ex* P. Wilson	Cidreira	Verbanaceae	Leaves	Abortifacient; Menstrual cramps	NI
	*Ruta graveolens* L.	Arruda	Rutaceae	Leaves; Flower	Menstrual cramps;	NI
Magalhães et al. (2022)	*Adiantium capillus-veneri* L.	Avenca	Pteridaceae	Leaves; Steam	Postpartum	Infusion
	*Plantago major* L.	Transagem	Plantaginaceae	Leaves	Ovarian infection	NI
	*Vitex sp.*	Jurema-de-cabloco	Lamiaceae	Leaves; seeds	Infertility; Controlling the menstrual period; Alleviating Menopausal Hot Flashes;	NI
Oliveira et al. (2014)	*Lippia origanoides* Kunth	Salva-de-marajó	Verbenaceae	Leaves; Aerial parts	Menstrual cramps; Pós-parto.	NI
Rodrigues (2007)	*Oxalis physocalyx* Zucc. *ex* Progel	Azedinha	Oxalidaceae	Whole plant	Abortifacient	Juice
	*Strychnos pseudoquina* A. St.-Hil	Quina	Loganiaceae	Leaves; Bark	Abortifacient	Decoction
Yazbek et al. (2019)	*Ageratum conyzoides* L.	Erva-de-São-João	Asteraceae	Leaves	Controlling the menstrual periodl	Maceration

## Discussion

Quilombola women experience in their daily lives the marks of exclusion and violence arising from prejudice against their skin color, culture and gender.^16^ These various forms of violence directly influence their health-disease process.^16^ Historically, the black population has been subjected to precarious health and welfare conditions, with the addition of Quilombola spaces being located in marginalized and rural environments, distancing these subjects from health services.^16^

Although these women transit in this process of exclusion, they have a leading role in their territories.^17^ They are responsible for the protection and rescue of ancestral knowledge that allowed the perpetuation of quilombos.^17^ Knowledge is passed down between generations, from mother to daughter, about the protection of the environment, food and health.^17^

Thus, in relation to gender, women are identified as the main holders of knowledge about medicinal plants.^18^^,^^19^ In Quilombola communities, it is common for women to dominate this knowledge, because most women are responsible for preparing home remedies and taking care of family health.^18^^,^^19^

The historical process of the construction of Quilombola spaces and their spatial arrangements allowed this population to develop knowledge about the medicinal properties of plants for the care of women.^20^ This fact explains the plurality of plants cited in the articles studied, totaling 44 plant species found in seven communities, with results like the study,^20^ in which the use of medicinal plants by Quilombola women in communities in the recôncavo of Bahia was investigated. They found 40 species reported for various therapeutic purposes, including relief from menstrual cramps, vaginal inflammation as well as menopause.

The plants found in this bibliographic research were pointed out for the care of the female reproductive system. The uterus is considered by these women as a sacred entity and responsible for the balance of general health.^20^^,^^21^ In this way, the care of these subjects and the appreciation of the puerperium process are understood, respecting the time-space of at least one month for the return of labor activities and 45 days for the return of sexual relations.^21^

The desire and care for puerperal women allow the construction of knowledge of medicinal plants in the care of inflammatory processes of the parturient woman/puerperium and postpartum pain, among them: white onion (*Allium ascalonicum* L.), carrapicho (*Bidens pilosa* L.), pega pinto (*Boerhaavia coccinea* Willd.), and cinnamon (*Cinnamomum zeylanicum* Breyn).^10^

The reported plants can also act on other aspects of women's sexual health, including menstrual regulation,^10^^,^^11^^,^^15^ stimulating uterine contraction,^10^ abortifacient^10^^,^^13^^,^^14^ and in menopause.^11^

Menstruation for Quilombola women becomes a tool of social control.^17^ Blood is seen as impure, making it impossible for them to carry out agricultural activities to avoid contamination of the soil, rivers and crops.^17^ Thus, there is a limitation of the economic activity of these women.^17^ Among the plants used for menstrual regulation, the following were cited: spearmint (*Mentha viridis* L.)^10^ and St. John's wort (*Ageratum conyzoides* L.).^15^

Quilombola women believe that medicinal plants are more efficacious when compared to pharmaceutical drugs.^22^ Thus, the use of these plants becomes frequent to treat signs and symptoms that compromise their health. It is considered that there is faster relief when resorting to a natural treatment.^22^

Although there is a process of expansion of assistance respecting the principles of SUS, some women need to resort to midwives, due to the distance between Quilombola communities and health services. These women are heirs to ancestral knowledge and are responsible for performing safe delivery, the first hygiene of these children and the care of the puerperal woman. Midwives are also called to care for abortion, being in charge of performing curettage. In this study, some of the species cited for cleaning the uterus were: artimijo (*Artemisia vulgaris* L.), and macela (*Chrysanthemum parthenium* L. Bernh).^10^

From the data presented, it is clear that the connection with nature made it possible to learn and improve practices based on traditional knowledge that accompany the dynamics of health care of traditional peoples, especially those of African origin in Brazil.^23^ Over the years, Quilombola populations have been gaining recognition for the conservation of socio-cultural and economic traditions, in order to use the land to maintain their material and immaterial goods and for practices to continue well-being and health.^24^^,^^25^

Knowledge about the forms of health care performed by Quilombolas is relevant as it allows rethinking care practices and health promotion strategies, in view of the specificity of quilombola women, focusing on providing integrated care within the scope of SUS.^24^^,^^25^

## Conclusion

Although there is a correlation between racial prejudices and the fragility of health care, education and information in these communities, it is observed that women play a fundamental role as holders and disseminators of knowledge about the use of medicinal plants in women's healthcare.

The diversity of herbs mentioned shows the deep connection between nature and health, highlighting the importance of preserving and valuing this cultural heritage. The valorization and recognition of this ancestral knowledge contribute not only to women's healthcare, but also to the strengthening of identity and the recovery of the cultural roots of these communities.

## Declaration of competing interest

The authors declare no conflicts of interest.
